# Game-related statistics that discriminate winning from losing in NCAA Division-I men's basketball

**DOI:** 10.3389/fspor.2024.1387918

**Published:** 2024-05-22

**Authors:** Dimitrije Cabarkapa, Damjana V. Cabarkapa, Andrew C. Fry

**Affiliations:** Jayhawk Athletic Performance Laboratory—Wu Tsai Human Performance Alliance, Department of Health, Sport and Exercise Sciences, University of Kansas, Lawrence, KS, United States

**Keywords:** sport, performance, analysis, shooting, rebounding, assists, turnovers

## Abstract

The purpose of the present study was to examine differences in game-related statistics between winning and losing game outcomes and determine which performance parameters have the greatest impact in classifying winning from losing game outcomes at the National Collegiate Athletic Association (NCAA) Division-I men's basketball level of competition. The data scraping technique was used to obtain publicly available data over a 2018–2019 season span. The total number of games examined was 5,147. Independent *t*-tests were used to examine statistically significant differences between winning and losing game outcomes, while a full model discriminant function analysis was used to determine the relative contribution of each game-related statistic and its ability to classify winning from losing game outcomes (*p* < 0.05). Alongside scoring a greater number of points at the end of the game, the findings of the present study indicate that winning teams: (a) attempted and made more field goals, three-point, and free-throw shots, (b) accumulated more defensive and total rebounds, assists, steals, and blocks, (c) had fewer turnovers and personal fouls, and (d) secured greater field goal, three-point, and free-throw shooting percentage. Moreover, the top three performance parameters discriminating winning from losing game outcomes were field goal percentage, defensive rebounds, and assists, accounting for 16.8%, 12.2%, and 12.0% of the total percentage of explained variance, respectively (i.e., 41.0% combined). Overall, these findings support the expected roles of offensive and defensive game-related statistics and provide further insight into how they work together to optimize the chances of securing the desired game outcome.

## 1 Introduction

Basketball is one of the most popular international sports in which each player, regardless of playing position (i.e., guard, forward, center), needs to possess fundamental basketball-specific skills such as passing, dribbling, shooting, rebounding, and the ability to play defense. Quantitative analysis of game-related statistics is commonly used to objectively assess the individual player and overall team performance efficiency (e.g., field goal percentage, assists, turnovers, defensive rebounds). This information may allow coaches and sports scientists to adequately plan training regimens and identify areas for basketball skill-related improvements that are of critical importance for securing the winning game outcome (1, 2).

A considerable amount of scientific literature has been focused on examining game-related statistics that discriminate winning from losing game outcomes on a professional level of basketball competition (3–9). When examining 306 regular season games played in ACB Spanish Basketball League, Garcia et al. (6) found that winning teams dominated in defensive rebounds, assists, and successful two- and three-point field goals. Moreover, defensive rebounds and assists were found to be two performance parameters that differentiated winning from losing game outcomes regardless of the game location (i.e., home vs. away) (10). Similar observations were made by Trninic et al. (11) when analyzing games played during the post-season competitive period during European Club Championship (i.e., Final Four). Game-related statistic with the greatest discriminating power in securing the desired game outcome was defensive rebounds, followed by field goal and free-throw shooting percentage (11). Also, defensive rebounds were found to be a key performance parameter differentiating between winning and losing teams during balanced games (i.e., final score ≤12 points), and successful two-point field goals, defensive rebounds, and assists during unbalanced games (i.e., final score >12 points) (9). Furthermore, a recently published study examined game-related statistics at the National Basketball Association (NBA) competitive level across a three-season span (2016–2019) (3). The findings revealed that field goal percentage and defensive rebounds were two performance parameters with the greatest percentage of explained variance capable of discriminating winning from losing game outcomes during both regular and post-season (3).

Unlike the previously mentioned research reports, quantitative analysis of game-related statistics at the amateur and/or collegiate level of basketball competition remains largely underexamined. To the best of our knowledge, only a couple of studies attempted to address this issue (12, 13). Lorenzo et al. (12) found that turnovers and assists were two performance parameters distinguishing winning from losing teams in the Under-16 European Championship in close games (i.e., final score difference ≤9 points) and two-point field goals and defensive rebounds in balanced games (i.e., final score difference between 10 and 29 points). In addition, Conte et al. (13) used a magnitude-based approach to analyze differences between winning and losing teams at the National Collegiate Athletic Association (NCAA) Division-I men's basketball level of competition during the 2013–14 season across 20 close games (i.e., final score difference ≤9 points). The findings revealed that winning teams were likely to display a higher three-point shooting percentage and a greater number of defensive rebounds, and were very likely to attempt and make more free-throw shots (13).

Therefore, to bridge a gap in the scientific literature and provide coaches and sports scientists with information that can be used to adequately plan training regimens and identify areas for basketball skill-related improvements, the purpose of the present study was to examine differences in game-related statistics between winning and losing game outcomes and determine which performance parameters have the greatest impact in classifying winning from losing game outcomes at the NCAA Division-I level of competition.

## 2 Materials and methods

### 2.1 Data source and procedures

Data scraping technique was used to obtain publicly available game-related statistics for NCAA Division-I men's basketball 2018–2019 competitive season from https://stats.ncaa.org website (i.e., contest - scoreboard - box score) via ParseHub software (North York, ON, Canada). This research report represents a follow-up analysis based on a previously published manuscript focused on examining the impact of home-court advantage on the same level of competition (14). The following 18 variables (i.e., team averages) were acquired from the box scores across 5,147 games: field goals made (FGM), field goals attempted (FGA), field goal shooting percentage (FG%), 3-point shots made (3PM), 3-point shots attempted (3PA), 3-point shooting percentage (3P%), free-throws made (FTM), free-throws attempted (FTA), free-throw shooting percentage (FT%), offensive rebounds (ORB), defensive rebounds (DRB), total rebounds (TBR), assists (AS), steals (ST), blocks (BL), turnovers (TO), personal fouls (PF), and points (PTS). Due to the public availability of the NCAA Division-I game-related statistics, the Institutional Review Board's approval for conducting this project was not needed.

### 2.2 Statistical analysis

Shapiro-Wilk test and Q-Q plots were used to assess that the assumption of normality was met. Descriptive statistics, means and standard deviations (x¯ ± SD), were calculated for each dependent variable. Independent *t*-tests were used to examine statistically significant differences between winning and losing game outcomes. Cohen's *d* was used to calculate the measure of effect size (i.e., *d* = 0.2 small, *d* = 0.5 moderate, and *d* = 0.8 large). A full model discriminant function analysis was used to examine the magnitude of the relative contribution of each game-related statistic and the ability to classify winning from losing game outcomes. To avoid the issue of multicollinearity, FTM, FGM, 3PM, TRB, and PTS were not included in the discriminant function analysis (e.g., 3P% = 3PM/3PA × 100%) (3). In addition, the intra-class correlation coefficient of 0.98 demonstrated the perfect reliability of the data scraping technique used to gather game-related statistical parameters across 100 randomly selected games. Statistical significance was set *a priori* to *p* < 0.05. All statistical analyses were completed with SPSS (Version 26.0; IBM Corp., Armonk, NY, USA).

## 3 Results

Statistically significant differences between winning and losing game outcomes were found for every game-related statistic (all *p* < 0.001), except ORB (*p* = 0.859). Besides scoring more PTS, winning teams had more FGM, FGA, 3PM, 3PA, FTM, FTA, DRB, TRB, AS, ST, BL, less TO and PF, and greater FG%, 3P%, and FT% (see Table 1).

**Table 1:** Descriptive data (x¯ ± SD) for game-related statistical parameters between the winning and losing game outcomes.

Game-related statistics	Losing teams	Winning teams	Effect size
Field goals made	23.5 ± 4.2	27.3 ± 4.5^†^	0.873 (L)
Field goals attempted	57.4 ± 6.9	58.1 ± 7.2^†^	0.099 (S)
Field goal percentage	40.6 ± 6.2	47.7 ± 6.3^†^	1.136 (L)
3-point shots made	7.0 ± 2.9	8.4 ± 3.2^†^	0.458 (M)
3-point shots attempted	22.1 ± 5.8	22.7 ± 5.9^†^	0.102 (S)
3-point shot percentage	30.9 ± 9.9	37.8 ± 10.2^†^	0.687 (M)
Free-throw shots made	11.8 ± 5.3	15.0 ± 6.0^†^	0.565 (M)
Free-throw shots attempted	17.2 ± 7.0	20.8 ± 7.7^†^	0.489 (M)
Free-throw shot percentage	68.6 ± 13.6	72.0 ± 11.5^†^	0.270 (S)
Offensive rebounds	9.9 ± 3.9	9.9 ± 3.8	0.000 (S)
Defensive rebounds	23.2 ± 4.5	27.1 ± 4.9^†^	0.829 (L)
Total rebounds	33.2 ± 6.1	37.0 ± 6.3^†^	0.613 (M)
Assists	11.5 ± 3.7	14.9 ± 4.4^†^	0.836 (L)
Steals	5.7 ± 2.7	6.7 ± 2.9^†^	0.357 (S)
Blocks	2.9 ± 2.4	3.9 ± 2.6^†^	0.399 (S)
Turnovers	13.6 ± 4.2	12.3 ± 3.8^†^	0.325 (S)
Personal fouls	18.8 ± 4.6	16.7 ± 4.2^†^	0.477 (M)
Points	65.8 ± 10.6	78.0 ± 10.7^†^	1.146 (L)

^†^Statistically significant when compared to the losing team (*p* < 0.05). S, small effect size; M, moderate effect size; L, large effect size.

A full-model discriminant function analysis was statistically significant [Λ = 0.440, X^2^(13)_ _= 8,694.84, *p* < 0.001] and capable of correctly classifying winning from losing game outcomes in 87.8% of cases. See Table 2 for standardized discriminant function coefficients, percentage of explained variance, and percentage of the total variance.

**Table 2:** Standardized discriminant function coefficients and percentage of explained and total variance for game-related statistical parameters.

Game-related statistics	Standardized coefficients	Percentage of total variance	Percentage of explained variance
Field goal percentage	0.513	19.1	16.8
Defensive rebounds	0.372	13.9	12.2
Assists	0.369	13.7	12.0
3-point shot percentage	0.314	11.7	10.3
Free-throw shots attempted	0.225	8.4	7.4
Personal fouls	0.210	7.8	6.8
Blocks	0.160	6.0	5.3
Steals	0.150	5.6	4.9
Turnovers	0.143	5.3	4.6
Free-throw shot percentage	0.122	4.5	4.0
Field goals attempted	0.045	1.7	1.5
3-point shots attempted	0.042	1.6	1.4
Offensive rebounds	0.002	0.7	0.6
Total		100	87.8

## 4 Discussion

The findings of the present study indicate that winning teams at the NCAA Division-I level of men's basketball competition had more FGM, 3PM, FTM, FTA, DRB, TRB, AS, ST, and BL, less FGA, 3PA, TO, and PF, and greater FG%, 3P%, and FT%, when compared to losing teams. The top three performance parameters discriminating winning from losing game outcomes were FG%, DRB, and AS, accounting for 16.8%, 12.2%, and 12.0% of the total percentage of explained variance, respectively (i.e., 41.0% combined).

### 4.1 Role of shooting efficiency

Alongside acquiring more scoring opportunities (i.e., FGA, 3PA, FTA), winning teams were capable of making more shots (i.e., FTM, 3PM, FTM), which resulted in superior shooting efficiency (i.e., FG%, 3P%, FT%) and ultimately led to a greater number of PTS scored at the end of the game. Identical findings were observed by Csataljay et al. (5) when analyzing game-related statistics during a regular season competitive period at the highest level of professional basketball competition in Hungary. Winning teams attempted and made more free-throw, two- and three-point shots across all quarters when compared to the losing teams (5). In addition, similar observations were made by Gomez et al. (9) when examining teams competing at ACB Spanish Basketball League. While no significant differences were noted for shots attempted from the free-throw line, winning teams attained more successful and less unsuccessful two- and three-point shooting attempts across 306 games played during a regular season span (9).

While securing more FGA, 3PA, and FTA creates additional scoring opportunities, it is important to note that the percentage of the explained variance for each of these game-related statistics is notably lower when compared to FG%, 3P%, and FT%. The FG% alone accounted for 16.8% of the total percentage of explained variance, and when combined with 3P% and FT%, the ability of these performance parameters to classify winning from losing outcomes increased to 31.1%. The findings of the present study suggest that the ability of a team to successfully utilize each scoring opportunity has a greater impact on securing the desired game outcome than solely creating additional scoring opportunities. For example, if two teams have the same number of FGA at the end of the game, the one able to secure greater FG% (i.e., winning team 47.7%; losing team 40.6%) is likely to win, and when accompanied by a greater number of FGA, the winning probability may further increase. In a recently published study, Cabarkapa et al. (3) found that FG% was one of the top two game-related statistics differentiating between winning and losing game outcomes at the NBA level of basketball competition during both regular and post-season competitive periods (i.e., winning team 48.4%; losing team 43.6%). Moreover, Conte et al. (13) found that winning NCAA Division-I men's basketball teams are likely to show effective FG% (e.g., winning team 46.0%; losing team 42.8%) and higher 3P% (i.e., winning team 39.6%; losing team 34.3%). Overall, the aforementioned findings further emphasize the importance of shooting efficiency, regardless of the shooting distance, as one of the key performance parameters for securing the winning game outcome.

### 4.2 Role of defensive rebounds and blocks

Alongside superior offensive performance, the findings of the present investigation reveal that winning teams had superior defensive performance when compared to losing teams, characterized by a greater number of DRB, TRB, and BL. Moreover, DRB was found to be a game-related static with the second largest percentage of explained variance in classifying between winning and losing game outcomes (i.e., 12.2%). Our results are in agreement with previously published research reports emphasizing the importance of DRB for securing the desired game outcome on various levels of basketball competition (3, 4, 6, 7, 12). When investigating 20 close games (i.e., final score difference ≤9 points) during the 2013–14 NCAA Division-I season, Conte et al. (13) found that winning teams were likely to have a higher number of DRB. Also, while having a significant contribution during the regular season, DRB demonstrated as a game-related statistic in which winning teams dominated during the post-season competitive period in ACB Spanish Basketball League (6). Acquiring a greater number of DRB minimizes the overall number of scoring opportunities and second-point chances for the opposing team, hence, increasing the likelihood of securing the winning game outcome.

Another defensive game-related statistic by which winning teams in the present study were characterized was a greater number of BL. Ibanez et al. (15) have found that a greater number of BL, as an indicator of better inside defensive pressure, was one of the top three performance parameters determining the season-long success in the Spanish Basketball League (LEB1). Interestingly, it has been found that away teams receive more BL, most likely due to greater defensive pressure applied by the home team used to disrupt the opponent's offensive strategies (10, 16). Based on basketball regulations, a defensive player is rewarded with a BL only when the ball is deflected during a field goal attempt. However, it is important to note that every attempt to block a shot, even if it does not result in a deflected ball, drastically increases the difficulty of the field goal attempt for the opponent. Thus, when combined with a greater number of DRB, having better rim protection (i.e., more BL) creates an additional advantage in securing the desired game outcome at the NCAA Division-I men's basketball level of competition.

### 4.3 Role of offensive rebounds

Interestingly, no difference in ORB performance was observed between winning and losing game outcomes in the present study. These findings suggest that both winning and losing teams tend to pursue the same amount of second-point scoring opportunities. Similar results were observed on various levels of basketball competitions, such as the Under-16 European Championship, ACB Spanish Basketball League, and NBA (3, 6, 12). Csataljay et al. (17) have found that the ORB efficiency is primarily influenced by the number of players dedicated to performing this task. In modern basketball, almost every offense starts or at least ends up with the middle ball screen (i.e., two players on one side and one player on the other side standing outside of the three-point line), where a player drives a ball down the lane and tries to pass it to an open player for an open three-point shot. This offensive strategy minimizes the number of mid-range jump shots and emphasizes long-distance scoring opportunities that can yield more points. Also, by utilizing this offensive strategy, with three players standing on a perimeter, it is difficult to pursue ORB. While further research is warranted on this topic, it is likely that all teams are utilizing similar offensive strategies, where 3–4 players are returning back on defense after an attempted field goal to prevent the opponent's fast-break scoring opportunities (3). Thus, based on these findings, we may assume that modifying offensive strategies and dedicating more players in pursuit of ORB may cause changes in other game-related statistics (e.g., a decrease in DRB for the opposing team) that could potentially influence the final game outcome.

### 4.4 Role of assists and steals

Winning teams at the NCAA Division-I men's basketball level of competition demonstrated better tactical discipline, characterized by a greater number of AS and ST, and fewer TO and PF. Based on basketball regulations, AS is defined as a pass that directly leads to a scored field goal (18). More AS indicates better team cohesion and implies a better decision-making process as it minimizes chances for TO and/or ST for the opposing team (6, 18). Also, a greater number of AS is one of the factors that allows a team to find better/uncontested scoring opportunities and ultimately attain better FG% (3). Our results indicate that the AS was one of the top three performance parameters in classifying winning from losing game outcomes, accounting for 12.0% of the total percentage of the explained variance. Similar observations were made by Trninic et al. (11) when examining the difference between winning and losing teams in European Club Championship tournaments (i.e., Final Four) across eight years span. The winning teams demonstrated better offensive control and were able to find more open-shot scoring opportunities (i.e., indicating a greater number of AS) that led to a lower number of TO and ultimately resulted in increased shooting efficiency (11). Moreover, fewer PF committed results in less FTA and ultimately minimizes the number of uncontested scoring opportunities for the opposing team. Previous research has found that expert players are capable of maintaining a better defensive intensity when compared to amateur players (19). In a similar manner, we can assume that winning teams had better defensive performance and were capable of minimizing the number of handicapped positions where PF needed to be committed in order to prevent the opponent from creating a scoring advantage and/or scoring an uncontested field goal.

### 4.5 Limitations

While these findings provide additional insight into game-related statistics associated with securing winning game outcomes at the NCAA Division-I men's basketball level of competition, this study is not without limitations. It should be acknowledged that data scraping has its limitations regarding the accuracy of the data obtained from publicly available sources. The game location (i.e., home vs. away), playing position, and the number of minutes played by each player was not included in the present analysis, which are factors that need to be considered and warrant further investigation. Also, future research should focus on examining if the observed findings apply to different levels of basketball competition (e.g., NCAA Division-II).

## 5 Conclusion

In conclusion, the winning teams at the NCAA Division-I level of men's basketball competition had more FGM, FGA, 3PM, 3PA, FTM, FTA, DRB, TRB, AS, ST, BL, less TO and PF, and greater FG%, 3P%, and FT% when compared to losing teams. The top three performance parameters discriminating winning from losing game outcomes were FG%, DRB, and AS, accounting for 16.8%, 12.2%, and 12.0% of the total percentage of explained variance, respectively (i.e., 41.0% combined). Overall, these findings support the expected roles of offensive and defensive game-related statistics and provide further insight into how they work together to optimize the chances of securing the desired game outcome.

## Data Availability

Publicly available datasets were analyzed in this study. This data can be found here: https://stats.ncaa.org.
